# Lessons learned obtaining informed consent in research with vulnerable populations in community health center settings

**DOI:** 10.1186/1756-0500-5-624

**Published:** 2012-11-07

**Authors:** Heather E Riden, Kya N Grooms, Cheryl R Clark, Laura R Cohen, Josh Gagne, Dora A Tovar, Mark J Ommerborn, Piper S Orton, Paula A Johnson

**Affiliations:** 1 Division of Women’s Health, Brigham and Women’s Hospital, 75 Francis Street, Boston, Massachusetts, USA; 2 Connors Center for Women’s Health and Gender Biology, Brigham and Women’s Hospital, 75 Francis Street, Boston, Massachusetts, USA; 3 Center for Community Health and Health Equity, Brigham and Women’s Hospital, 1620 Tremont Street, Boston, Massachusetts, USA; 4 Division of General Medicine and Primary Care, Brigham and Women’s-Faulkner Hospitalist Program, 1620 Tremont Street, Boston, Massachusetts, USA; 5 Harvard Medical School, 25 Shattuck Street, Boston, Massachusetts, USA; 6 Survey & Data Management Core, Dana-Farber Cancer Institute, 450 Brookline Avenue, Boston, Massachusetts, USA

**Keywords:** Community-based participatory research, Patient selection, Health status disparities, Informed consent, Poverty, Vulnerable populations

## Abstract

**Background:**

To improve equity in access to medical research, successful strategies are needed to recruit diverse populations. Here, we examine experiences of community health center (CHC) staff who guided an informed consent process to overcome recruitment barriers in a medical record review study.

**Methods:**

We conducted ten semi-structured interviews with CHC staff members. Interviews were audiotaped, transcribed, and structurally and thematically coded. We used NVivo, an ethnographic data management software program, to analyze themes related to recruitment challenges.

**Results:**

CHC interviewees reported that a key challenge to recruitment included the difficult balance between institutional review board (IRB) requirements for informed consent, and conveying an appropriate level of risk to patients. CHC staff perceived that the requirements of IRB certification itself posed a barrier to allowing diverse staff to participate in recruitment efforts. A key barrier to recruitment also included the lack of updated contact information on CHC patients. CHC interviewees reported that the successes they experienced reflected an alignment between study aims and CHC goals, and trusted relationships between CHCs and staff and the patients they recruited.

**Conclusions:**

Making IRB training more accessible to CHC-based staff, improving consent form clarity for participants, and developing processes for routinely updating patient information would greatly lower recruitment barriers for diverse populations in health services research.

## Background

Though the National Institutes of Health Revitalization Act of 1993 mandated the recruitment of minority populations into federally-funded research studies [1], minority and low-income women are still underrepresented in medical and health services research [2]. There is an essential need for new and effective strategies to increase participation of minority and low-income populations in medical research.

### Current evidence base

Recent literature suggests that challenges to the inclusion of diverse participants in research are experienced by potential research participants as well as research investigators and staff. Known barriers from the perspective of potential participants include: distrust of the research community [2-14]; lack of knowledge or awareness of medical research [3,6,8,12,14-16]; concerns over confidentiality and privacy [10,11,14,17,18]; fear that participation in a research study could result in a loss of health services or deportation due to immigration status [10,11]; and an ineffective informed consent process [3,11,19,20]. Barriers faced by research investigators and staff may include: the difficulty in “locating” potential subjects [18,21]; investigators’ personal biases [5-7,12]; and cultural differences between investigators and participants [3,11,14,19].

### Context of the current study

To overcome recruitment barriers, increasingly, there are opportunities for community health centers (CHCs) and academic medical centers to partner to improve recruitment of minority or vulnerable populations into biomedical, health services, and community participatory research [22]. Community health centers play a critical role in delivering care to diverse populations. Community-based participatory research partnerships between CHCs and academic institutions may enhance recruitment of diverse populations, and facilitate a culturally sensitive approach to medical research [23]. While academic medical institutions can provide expertise in study design and research processes, CHCs can offer important knowledge and cultural contexts of their patient populations that may enhance recruitment.

Through a partnership between an academic medical institution and five CHCs in the greater Boston area, we have recently had success enrolling a diverse population of women in the Brigham and Women’s Hospital Advancing Systems Improvements to Support Targets for Healthy People 2010 (ASIST) study, an investigation of the impact of Massachusetts health care reform on the use of preventive health screening by low-income women. Community health center staff members were instrumental in obtaining the consent of over 1,200 patients to participate in the study. Participants were low-income women from diverse ethnic and racial backgrounds. Recruitment consisted of contacting participants primarily by phone, and also during outpatient clinic visits, to request permission for ongoing medical record access and review, and permission to review participants’ insurance claims from data obtained from the state. CHC staff described the study objectives to potential participants, described study risks and benefits, and reviewed formal IRB-designed consent materials with participants before requesting participant consent by phone or in person. The process of recruiting and obtaining the informed consent of minority and low-income women presented numerous challenges for which unique solutions were devised by CHC staff.

To “unpack” the challenges and lessons learned from a successful recruitment effort in CHC settings, we have conducted a qualitative study in which we interviewed health center staff regarding their experiences, challenges, and successes in recruiting and obtaining the consent of a diverse group of women to participate in research. We explored CHC staff perspectives on what they felt were patients’ barriers and facilitators to participating in research, and staff strategies for overcoming these barriers. We also explored CHC staff motivations for, and experiences in, partnering with an academic center in a research project. The objective of the present study is to describe themes related to the barriers in the recruitment of diverse populations in medical and health services research, and to describe specific details on the facilitating factors and strategies used to address these challenges from the perspective of CHC staff. A better understanding of the recruitment process from the perspective of CHC staff may help develop more effective strategies for enrolling diverse populations in biomedical research studies.

### Research questions

A. Explore CHC staff perceptions and experiences with enrollment processes that facilitated and posed barriers to patient recruitment.

B. Understand CHC perceptions and experiences with processes of the community-academic research partnership that facilitated and impeded patient recruitment.

## Methods

### Study design, sample, and ethical approval

We interviewed CHC staff members, directors and administrators who were involved in the recruitment and enrollment process for the medical record review study. We attempted to obtain a complete census of the CHC staff who participated in study recruitment activities. All thirteen individuals who played a role in the medical record review study enrollment activities were invited by the parent ASIST study investigators to participate in the interviews. Of these thirteen, ten individuals agreed to be interviewed, and three individuals did not respond to requests for an interview. In February and March 2010, a single trained interviewer was hired to conduct one-on-one in-person interviews using two separate semi-structured interview guides. The interview guide was designed to probe themes in the literature related to recruitment barriers and facilitators. The first guide was used in interviews with health center staff who directly obtained consent from women in the study (See Appendix 1). The second guide was used in interviews with staff members who coordinated their health center’s participation in the enrollment process (See Appendix 2). The trained interviewer used a “semi-structured” format allowing for follow-up questions, probing by the interviewer, and free flowing feedback from the staff members to allow unanticipated themes to emerge. The content of the interviews focused on six relevant areas designed to explore CHC perspectives on enrollment processes (Research question A), and academic-community partnership factors that may influenced recruitment (Research question B). These specific topics included: 1) the challenges and successes in obtaining informed consent from the patients (question A); 2) their perceptions of interactions with potential subjects (question A); 3) the barriers encountered during enrollment (question A); 4) perspectives on enrollment logistics and center-level support from the CHCs and the academic medical institution (question B); 5) rewards of the partnership and lessons learned (question B); and 6) the overall experience participating in the medical record review study and recommendations for future research (question B). Interviews ranged from thirty minutes to two hours. Interviews were audio recorded and professionally transcribed. Interviewees received $25 in compensation. Interviewers obtained written informed consent from study participants. The Partners Human Research Committee approved this study.

### Qualitative data coding and analysis

The transcribed interview data were analyzed first by a single coder, according to a standard two-stage coding process. Level 1 offered structural coding, while Level 2 offered thematic coding. Structural coding was used to provide a “code,” or annotation, to link each planned interview question and its associated interviewer probes with the participant response text. Structural codes were then used to inform thematic analysis. A single independent analyst with expertise in cultural anthropology, who was *not* involved in the parent study or recruitment processes, first identified emergent themes in the analysis of the structural codes. The analyst’s initial thematic coding was then validated by four reviewers from the academic team of the parent study, who have expertise in public health, epidemiology, and program evaluation. The four reviewers examined the transcript and used an interactive process to develop consensus on central themes and sub-themes that emerged from the structural and thematic codes. The review process was both deductive (alert to themes detailed in the empiric literature) and inductive (open to novel themes that re-occurred independently across interviewees, but were not previously emphasized in the literature). Structural and thematic coding was performed with an ethnographic data management software program, NVivo (version 8 - QSR International). The NVivo program used an organizer indexing system to: code, categorize, search, and retrieve themes; attach analytical memos; and create conceptual relationship networks in textual data that has been taxonomically coded.

## Results

### Demographics and themes

Of the ten interview participants, five obtained consent from patients to participate in the medical record review study and five coordinated CHC activities related to study enrollment. Interviewees held varied positions within the CHCs, such as medical assistants, research assistants, executive directors, directors of interpreter services, and nurse care coordinators. In some cases, interviewees were research assistants specifically hired by the CHCs to enroll women. While these research assistants did not have prior relationships with patients, they contacted patients on behalf of the CHCs, while located on-site at the CHC. Three interviewees were black (30%), four were white (40%), two were Hispanic/Latina (20%), and one identified as white and Latina (10%). Importantly, CHC staff often spoke the native languages of their diverse patient population. The demographic characteristics of the patient population recruited by CHC staff are listed in Table 1.

**Table 1:** Characteristics of Women Enrolled in the ASIST Study

	**N = 1214**
**Race/Ethnicity**	
White, non-Hispanic	345 (28)
Black, non-Hispanic	210 (17)
Hispanic	534 (44)
Asian	110 (9)
Other, Unknown	15 (1)
**Educational attainment**	
Less than high school	502 (41)
High school graduate	332 (27)
Any college	304 (25)
Unknown	76 (6)
**Median household income, $**	$10,129

*Note:* Figures are presented as N (%), unless otherwise noted.

Summarized in Table 2, the five major themes that emerged during the interviews included: 1) challenges staff faced during the enrollment process; 2) staff motivation to participate in the medical record review study and positive experiences gained by participating; 3) patients’ perceived motivation to enroll in the medical record review study; 4) patients’ expressed concerns related to privacy and disclosure of personal medical records; and 5) challenges related to Institutional Review Board (IRB) procedures regarding training for CHC staff and informed consent forms for patients.

**Table 2:** Themes from Interviews with Community Health Center Staff

1.	Health center staff faced challenges during the enrollment process related to:
	Difficulty locating accurate patient contact information.
	Difficulty reaching women when accurate contact information was available.
	Understanding the cultural context of informed consent the patient population.
	Conducting research in a clinical setting.
	Difficulty locating accurate patient contact information.
	Difficulty reaching women when accurate contact information was available.
	Understanding the cultural context of informed consent the patient population.
	Conducting research in a clinical setting.
2.	CHC staff was motivated to assist in the chart review study in order to use the results to better serve their patients and community.
3.	Trust in, and relationships with, CHCs and health center staff motivated patients to participate.
4.	Patient concerns centered on privacy and disclosure of their medical records.
5.	IRB procedures posed study barriers that included training for staff and consenting patients:
	Complexity of the IRB training requirements for health center staff.
	Complexity of consent form length, language, literacy level and cultural concerns of patients.
	

### Staff challenges during the enrollment process

#### Challenges reaching women

The two greatest barriers involved in obtaining consent from women were the difficulty locating accurate contact information and the difficulty reaching women by telephone or in person. The medical record review study sought to enroll women who received cancer and cardiovascular disease screenings funded by the Massachusetts Department of Public Health Women’s Health Network program for low-income women during a defined time period prior to the implementation of Massachusetts health care reform. Enrollment began two years following the implementation of health care reform. During the enrollment period, accurate contact information was not available for 30% of the 2,903 women identified as eligible to participate in the medical record review study. Inaccurate contact information accounted for 51% of non-participation in the medical record review study (Table 3). This challenge is articulated by a respondent:

**Table 3:** Reasons Women Did Not Enroll in the ASIST Study, by Race/Ethnicity

	**Non-participant Total**	**Incorrect Contact Information**	**Correct Contact Information, No Response**^**a**^	**Declined**
**Race/Ethnicity**				
White, non-Hispanic	612	321 (52)	213 (35)	78 (13)
Black, non-Hispanic	265	143 (54)	91 (34)	31 (12)
Hispanic	639	329 (51)	275 (43)	35 (5)
Asian	117	48 (41)	49 (42)	20 (17)
Other, Unknown	56	22 (39)	26 (49)	8 (14)
**Total**	1689	863 (51)	654 (39)	172 (10)

*Note:*^a^Coded after 3 calls were made to contact eligible women. Figures are presented as N (%) of the non-participant total.

“[The biggest challenges were]…the nomadic nature of the patients we were trying to reach. Most of them had moved, changed phone numbers…”

This theme is echoed by another respondent:

“So many of them were not reachable that you could go through twenty names in five minutes, because the numbers were all disconnected.”

Even when health center staff members were able to locate accurate contact information, reaching women to request their enrollment in the study was difficult. Health center staff were able to reach 48% of eligible participants (n = 1,386). According to one respondent:

“…these women, they tend to work several jobs, so finding a right time to give them a call was a barrier for us.”

Health center staff members employed several strategies to reach women, including checking the clinic schedule for upcoming appointments for eligible women, varying the time of day when they called, and calling at the beginning of a month as some recruiters found it to be more likely that phones were disconnected later in the month. When potential participants were reached, 88% (n = 1,214) consented to participate in the study. Only 172 women actively declined when asked to participate (Table 3).

#### Cultural competency during recruitment process

Several respondents emphasized the importance of being aware of language, culture and literacy when interacting with patients. Respondents noted that some patients required the input of patients’ families or other trusted sources prior to consenting to participate in research. This was particularly true for non-English speaking patients. One respondent noted:

“There are a couple of the calls we made … through [the] interpreter, you know, that it was very hard. You know, they, didn’t want to talk to us, and, when they did they truly wanted other family members’ input and I don’t think it was because they didn’t understand; it’s because they truly felt they couldn’t make that decision on their own.”

A second staff member noted:

“Some of the older women wanted their daughter’s or younger sibling’s input. A couple of them said, “I need my husband’s input. …So, they would say, ‘You know, I think I understand you, but, would you explain it to my daughter, to see if it’s something I SHOULD do?’”

Another staff member commented:

“…there’s a little bit of a clash between the, maybe, Western medical idea about, you know, that the patient is the one that always needs to be informed and is always empowered to make the decision to consent? Um, there were occasions when, um, a patient comes to the health center consistently with their husband or their partner…and that’s the person that they kind of rely on to be the interface between them and the medical system. And if I had, you know, excluded that person and just talked to the woman… like, that kind of, I, um, don’t feel like she would have been comfortable, you know, saying yes. So I would, um… you know, I would… approach, I would speak to… to both of them, and show the consent form to both of them.”

#### Challenges conducting research in a clinical setting

Other challenges discussed by respondents included: the additional work created by assisting in the study; inadequate staffing; and a feeling that more time was needed for the enrollment process.

### Staff motivation and positive experiences

Interviewees were asked about their motivation for assisting in the medical record review study. Motivating factors included: a desire to learn from study results in order to better serve their patients and community, the scope of the study objectives, and the potential for impact at a national level. One respondent stated a desire:

“…to learn about the study and how we can use that information to better serve the…women in the community, which is an under-served population, and making sure they get access to care.”

Others reported that the potential to contribute to discussions of national health care reform policy by learning from the impact of Massachusetts’ health care reform on their patients was rewarding.

“…that folks…from Washington are taking a look at this study and…our little health centers…are actually part of the bigger picture, now.”

### Perception of patient motivators and trust

Respondents observed that patients were motivated to participate in the medical record review study because of their trust in, and connection to, the CHC and its staff. A respondent reflected:

“I think for us what really was key was the fact that those staff already had existing relationships with those patients. So they essentially put their trust in the staff.”

Another respondent felt that the number of women who enrolled was directly connected to health center staff being involved:

“I think it would have been a lot harder if, for example, like an outside organization had come in and just, you know, called all of our patients…That maybe would have been quicker, but I’m not sure we would have yielded the same amount of positive responses.”

### Perception of patient concerns

Patient concerns, as expressed to health center staff, centered on privacy and disclosure of medical records. Related to privacy concerns, respondents indicated that some patients, Hispanic/Latina women in particular, were concerned that their immigration status could be investigated and/or their eligibility for public programs could be at risk.

“…the word [for research] in Spanish and Portuguese is ‘investigation’ as opposed to ‘research’… So that led them to think that we’re doing some police or federal investigation…”

Lastly, some patients were unfamiliar with the concept of research in general, and differentiating participation in the medical record review study from direct receipt of services was a challenge:

“…a lot of them wanted to know if we would try to get them insurance, if we could help them with appointments… it was really difficult for them to understand the concept of a research study.”

### Challenges of IRB procedures

#### Challenges with human subjects training

All staff involved in the enrollment process were required to become certified in the ethical conduct of research by taking an online training program. Respondents reported that the course was difficult for CHC staff members who were not native English speakers and/or had no prior experience with research studies. One respondent stated:

“They [staff] come from a lot of different cultures, and they have different levels of English understanding… the IRB [course] was clearly written for more of an academic institution, or… research hospital setting, which is not a community health center.”

Health center staff also reported learning about research and IRB processes through their participation in the study. One respondent stated:

“It helped us to build our capacity a little bit so that if and when another opportunity comes along like this, we know… that we can do it, and we know a little bit about how to approach it the next time.”

#### Problems with consent forms

Every director/coordinator recommended that the IRB training requirements for staff be simplified. Respondents also felt that the consent forms were complicated for potential subjects, citing patient literacy as a barrier, and recommended simplifying the consent form. The consent form for the medical record review study was eleven pages long and used a template required by the institutional human subjects committee. Consent forms were written at a sixth-grade reading level, two grades lower than is standard for projects approved by our institutional review committee, and translated into ten languages. However, several respondents stated that the sixth-grade reading level consent form was written at too high a literacy level for their patients. One felt that its length raised concerns in some patients:

“I think that the length of the form, itself, that the actual overall length of it, I think that makes it kind of misleading as to how risky or involved the study is.”

Another interviewee recommended:

“Keep the process on the actual consent forms as simple as possible… as simple as the IRB will allow, so as not to frighten people.”

Respondents had several additional recommendations for conducting future research in CHC settings: 1) involve consenting staff early on in designing the informed consent process; 2) have a dedicated phone line for making calls and a quiet work space; 3) research phone numbers prior to beginning enrollment; 4) have a system to link patients to other studies taking place at the academic medical institution; and 5) develop clinical processes to flag patients with incorrect contact information so information could be updated with each patient encounter.

## Discussion

Our study was designed to explore challenges, successes, and lessons learned in a successful effort to recruit diverse and vulnerable patients to participate in research in CHC settings. Five themes emerged from interviews with CHC staff that provide insight into challenges and successful strategies for obtaining consent from diverse populations in CHC settings. Of these, we identified a novel theme rarely reported in the literature, that the IRB training process can pose major barriers to research participation for CHC staff. CHC staff indicated that the content of the standardized online training program for the ethical conduct of research was difficult to process and that the technical language in this training program was particularly inaccessible for CHC staff members who were non-native English speakers. Our study also found that the informed consent process may have presented a barrier for study participants as well. CHC staff indicated that the level of detail and disclaimers written into boilerplate institutional consent forms may have discouraged participation, and suggested a greater level of risk to participants than might have been warranted, given that the most likely risk of participating in our study was a loss of privacy. Third, CHC staff indicated that difficulty locating potential participants due to participants’ residential mobility and/or lack of up to date contact information was the greatest barrier to recruitment. When contact information was accurate, connecting with potential participants may have depended on whether phone service was active, or on finding a time of day when participants would be free at a given site of contact. Fourth, we found that CHC staff were highly motivated to participate in achieving the research study aims, and were willing to commit time to the project in order to learn about the impact of Massachusetts health care reform on populations they serve. Last, CHC center staff suggested that the success of the enrollment process was largely the result of having a diverse staff who had long-standing relationships with CHC clients that facilitated trust and culturally competent communication during recruitment.

Our findings on strategies used by ASIST CHC staff reflect some the suggested strategies to increase enrollment of minority and low-income populations reported in the literature. These strategies include: aligning the research study goals with the goals of the host community [2,10,11]; employing research staff who are a cultural, racial and/or linguistic match to potential subjects [2,6,8,10,11,16,18,19,24]; increasing the length of the enrollment period [24,25]; increasing cultural competence and awareness among research staff [5,6,19]; and having a clear and effective informed consent process that gives a balanced description of the study purpose and risks of the research to the participant [3,6,9,20]. To overcome the novel barrier we encountered related to the complexity of the training in the ethical conduct of research, the academic staff developed an “IRB training primer” to make the purpose and content of the standardized online training more accessible.

We note that the major challenge of obtaining accurate contact information and reaching CHC patients by phone has implications for providing quality clinical care as well as for equitable representation in research [26]. Specific to our diverse, low-income population, CHC staff suggest the following strategies may address the barriers associated with contacting and locating potential participants: create flexible hours for staff to allow for day and evening recruitment calls; call individuals at the beginning of the month when telephone service is more likely to be active (because payment is up-to-date or cell phone minutes are recharged); and update contact information at regular intervals, including at each encounter and between encounters if possible.

To address barriers related to informed consent, we suggest working with IRB committees to modify consent forms to ensure cultural literacy and communication of the appropriate level of risk. Further examination of the effectiveness and appropriateness of consent form language, related to community-based research, is warranted.

Similar to past research [2-14], we found patient trust in CHCs and their staff was an important theme that may have influenced patients’ decision to enroll. While enhanced trust supports the involvement of CHC staff in recruitment activities, it also has one potential pitfall to be avoided. Patient trust in health center staff may be so deeply motivating that informed consent could be obtained on that trust alone, and without the patient’s full understanding of the goals, risks, and benefits of the research study. Strategies to avoid this possible outcome should include consent forms written for the literacy level of potential participants and a script for health center staff that describes the study in easy to understand terminology. We suggest another option may include a two-step process where health center staff inform patients about the project, and then refer patients to independent study staff for enrollment, so that patients do not feel obligated to participate based on their existing relationship with the health center. In addition, other studies have suggested the effectiveness of using informed consent forms, in conjunction with in-person discussions between research staff and participants, to increase understanding and awareness of the consent process [9,20].

Finally, we found that challenges related to extending the mission of CHCs from patient care to conducting research could be overcome through investment in training and capacity building. Specifically, we found that academic partner support was needed to accomplish training and certification in the ethical conduct of human research, and CHC institutional support for health center staff was needed in order to decrease time and space constraints for research processes. CHC staff and administrators were willing to invest these resources and time due to their support of the central study aims.

A key limitation of this study is that we did not interview the women who enrolled into the medical record review study. Understanding the enrollment process from the patients’ perspectives could add important information on effective strategies in the recruitment of minority and low-income populations. However, we were able to obtain proxy information on women who were unlikely to consent to research. Another limitation of the study is that the staff experiences were limited to the recruitment and enrollment of female patients of low socioeconomic status in CHCs in a specific geographic area. Further research using other demographic groups is warranted to identify whether these themes are universal or specific to vulnerable populations.

## Conclusions

Our study identified novel challenges, as well as strategies to overcome barriers to recruiting low-income women into a medical record review study. The major themes that may impact future efforts to recruit in community settings relate to 1) IRB procedures, 2) efforts to reach mobile populations, and 3) the unique opportunity for academic medical centers and community health centers to partner to enhance patient recruitment. Efforts to minimize these challenges and to utilize population-specific strategies to stay connected to vulnerable groups are critical to the successful recruitment of diverse populations in CHC-based research.

## Appendix 1

### Appendix 1 health center staff interview consenting staff

Thank you for meeting with me today. The first thing I’d like to do is go over a consent form with you.

We’d like to hear your thoughts on several aspects of the ASIST study. I thought I’d give you a general overview of the areas we will focus on today, and then we can jump into the interview. First, we will talk about what it was like for you to consent women to participate in the study. Here we will focus on the consenting process. We will also talk about what it was like for you to participate in the study more generally, the specific challenges you faced and the lessons you learned.

Do you have any questions before we get started?

We’ll begin by talking about your experience with the consenting process.

SECTION 1 – Experiences Consenting – Here we are focusing on what they think happened during calls.

1. First I’d like to ask you about your involvement in the study. What role did you play for *health center name* on the ASIST study?

a. Can you take me through the process that you went through to enroll women into the study?

b. How consenting go? What was it like to consent women?

2. Could you tell me about a time when you were talking to a patient and you had trouble getting them to enroll?

a. In general, what do you think made women refuse?

b. Can you think of a specific time someone refused and the reasons they gave?

3. Could you tell me about a time when you were successful in enrolling a woman who you didn’t think would say yes at the start of the conversation?

a. In general, what do you think made women agree to participate?

b. Can you think of a specific time someone consented and the reasons they gave?

4. Are there any other examples of calls you made that stick out in your mind? Can you describe a few?

a. What were the biggest challenges?

b. Can you describe specific successes you had?

SECTION 2 – Participant Relationships and Concerns – Here we are focusing on specific things that happened.

1. >What types of questions did patients ask about the study when you called?

a. Are there any examples of concerns they expressed that stick out in your mind?

b. What reasons did women give for consenting, or not consenting?

2. Did you have existing relationships with most of the patients you called?

a. What was it like consenting and talking to women you knew well versus those you didn’t know as well?

b. What strategies did you use with women you knew well versus those you didn’t know as well?

c. What steps did you take to introduce yourself to women?

3. In this study we are asking for patients to release their records to an organization with which they do not have a relationship.

a. What were women’s common concerns and/or questions about sharing their private medical records?

b. How did you handle those questions and concerns?

SECTION 3 – Vulnerable Population Challenges

1. Now I will ask about consenting women on your lists.

a. Can you talk about the amount of time it took to reach women?

b. Which women were harder to reach? What about them made them difficult to reach?

c. Which were easier? What about them made it easy to reach them?

2. When you called to consent women, do you feel like you were always talking to the person who was able to make the decision to participate?

a. Did people tell you that they needed someone else’s input? How did this come up? Who did they want to talk about the study with?

b. Do you have a sense of which women this was relevant for?

3. Were consent forms adequate – in language, literacy, tone, readability?

a. What problems came up when reviewing the consent form with women?

b. Were the forms easy for women to understand?

SECTION 4 – Institutional Buy-In

1. How did you explain the study to women and discuss participating in the study?

a. Do you think your ability to explain the study impacted the consenting process? If so, how?

b. Was it difficult for you to describe the study goals?

c. If yes, do you feel like you now better understand the study? At what point in the process did you feel you really understood the study goals? What helped you gain this understanding?

2. Can you describe when and where you made calls or met with women to enroll them in the study?

a. Did you have enough time in your work day to consent women?

b. Were there any space constraints that affected your effort to consent women?

c. Were there any other constraints that affected your effort to consent women?

3. Do you feel like you received enough support and encouragement for the study from *health center name?*

a. How did *health center name* make it easier for you to do the consenting?

b. In what ways did you not receive enough support? What could they have done to help you more?

SECTION 5 – Challenges Summary

1. Here is a list of the types of challenges we have heard study staff mention regarding consenting.

Privacy – medical records

Patient trust

Patient/consenter relationship

Accurate list from DPH (criteria of study – age, pregnancy)

Ability to explaining the study

Old contact information

Time constraints

Space constraints

Health center buy-in

Support from BWH

Language barriers

Literacy concerns

a. Is there anything you would add to this list?

b. Is there anything on this list that was not an issue at *name of health center*?

c. Of these challenges, which stands out to you as the one that affected *name of health center* most?

d. *If the challenge they talked about in Section 1 Question 4 is on this list – ask*: When you look at this list, do you still consider *______* to be the greatest challenge?

e. How would you rank these topics from greatest to least challenging?

2. You identified ________ as the greatest challenge to participating in the study.

a. What did you do to address that challenge?

Is there anything else you would like to add about any of these areas that we haven’t already discussed? *(Skip if already discussed extensively)*

SECTION 6 – Rewards and Lessons Learned

We just spent time discussing different challenges, now I would like to ask about successes and lessons learned.

1. What motivated you to participate in ASIST?

a. What made you interested?

2. What did you find rewarding about participating in the ASIST study?

3. What did you learn during the course of the study?

a. Did you gain any new skills through participating? What were these skills?

4. What big picture lessons or take home points did you gain through participating in the study?

SECTION 7 – Study Participation and Future Research

1. Did participating in the study impact your relationship with patients?

a. If yes, how?

2. Did participating in the study give you any new insights into delivering care with the population of patients you serve?

Part of our hope with these interviews is that we will be able to provide information to future researchers on how to conduct these types of studies.

3. Are there any recommendations you would make to future researchers developing this kind of study?

d. What changes would have made the process easier for you?

e. Are there resources you would recommend future researchers provide to community health centers?

f. Would you be interested in participating in this type of research again? Why or why not?

4. Do you have any other comments or thoughts about participating in the ASIST study?

That wraps up the questions I have for you. Is there anything that we didn’t talk about that you would like to tell us?

Lastly, I would like to gather a few pieces of demographic information from you:

What is your title at *health center name*?

How long have you been employed at *health center name*?

What is your primary language?

What is your highest level of education?

What is your race/ethnicity?

Thank participant.

## Appendix 2

### Appendix 2 health center staff interview directors and coordinators

Thank you for meeting with me today. The first thing I would like to do is go over a consent form with you.

We would like to hear your thoughts on several aspects of the ASIST study. I thought I would give you a general overview of the areas we will focus on, and then we can jump in. First we will talk about what it was like for you to coordinate your health center’s effort to consent women to participate in the study. Here we will focus on the logistics of the consenting process. We will also talk about what it was like for you to participate in the study more generally; specific challenges you faced; and lessons learned.

Do you have any questions before we get started? Like I mentioned, we are going to start with the logistics of the consenting process.

SECTION 1 – Experiences Coordinating Consenting Process

1. First I would like to ask you about your involvement in the study. What role did you play for *health center name* on the ASIST study?

2. How were you involved in helping *health center name* prepare to consent women for the study?

a. What issues did you confront?

b. How did you work around those issues to enable the consenting process to begin?

c. What kind of issues were one-time problems that once sorted out, did not come up again?

d. How did you resolve those challenges? Are you satisfied with how they were resolved?

e. What kind of issues where ongoing problems that came up regularly?

f. How did you address those challenges?

3. How did transitions in staff and/or directors at *health center name* affect your experience in participating in the study?

a. If applicable: What was it like to enter the study after it had begun?

b. If applicable: What did you need to do to support ongoing efforts?

4. Can you describe a memorable challenge you faced in coordinating the consenting process?

5. Can you describe a memorable success you achieved when coordinating the consenting process?

SECTION 2 – Reaching a Vulnerable Population

1. From your perspective, what was it like for your staff to contact and reach women to participate in ASIST?

a. Were you surprised by any part of what needed to be done to reach women?

b. What challenges did you, or your staff, face in consenting women?

c. Which women were easier or harder to reach from your perspective?

d. What new insights about reaching your patient population were gained?

2. How did literacy and language barriers come up at *health center name* related to the study?

a. How adequate were the tools to deal with it?

3. Did any patients come to you directly to discuss the study?

a. What did they want to talk about?

b. Were there any complaints? If so, what was the patient’s concern?

SECTION 3 – Institutional Buy-In

1. What resources did your *health center name* have that enabled participation in the study?

2. What barriers did you face that made it difficult to participate in the study? (staffing, study tools, EMR, etc) *For North Shore staff, ask these questions twice, for Salem and for Lynn.*

a. Was it difficult to find staff to consent women for the study?

b. Was it difficult to find space for your staff to use when working on the study?

c. Were there other barriers you faced that made participation difficult?

d. What resources would have made participating in the project easier?

SECTION 4 – Challenges Summary

1. Here is a list of the types of challenges we have heard study staff mention regarding consenting.

Privacy – medical records

Patient trust

Patient/consenter relationship

Accurate list from DPH (criteria of study – age, pregnancy)

Ability to explaining the study

Old contact information

Time constraints

Space constraints

Health center buy-in

Support from BWH

Language barriers

Literacy concerns

a. Is there anything you would add to this list?

b. Is there anything on this list that was not an issue at *name of health center*?

c. Of these challenges, which stands out to you as the one that affected *name of health center* most? *If the challenge they talked about in Section 1 Question 5 is on this list – ask*: When you look at this list, do you still consider *______* to be the greatest challenge?

d. How would you rank these topics from greatest to least challenging?

2. You identified ________ as the greatest challenge to participating in the study.

a. What did you do to address that challenge?

3. What health center resources or other tools were you able to use in order to make consenting more efficient?

a. Were adjustments made to the consenting process after starting the study that improved the process?

b. What helped make consenting as efficient as possible?

c. What changes were made? *(This may include changing which staff at the health center contacted patients; the method by which patients were contacted, etc.)*

4. Is there anything else you would like to add about any of these areas that we haven’t already discussed? *(Skip if already discussed extensively)*

SECTION 5 – Rewards and Lessons

We just spent time discussing different challenges you and your staff faced, now I would like to ask about successes and lessons learned.

1. What motivated you to participate in ASIST?

a. What made you interested?

2. What did you find rewarding about participating in the ASIST study?

3. What did you learn during the course of the study?

a. Did you gain any new skills through participating? Such as?

4. What big picture lessons or take home points did you gain through your participation?

5. What do you think motivated your staff to participate?

a. What aspects do you think they found rewarding?

6. What do you hope the study outcomes will show?

SECTION 6 – Study Participation and Future Research

1. Did participating in the study impact your relationship with patients?

a. If yes, how?

2. Did participating in the study give you any new insights into delivering care with the population of patients you serve?

Part of our hope with these interviews is that we will be able to provide information to future researchers on how to conduct these types of studies.

3. Are there any recommendations you would make to future researchers developing this kind of study?

d. What changes would have made the process easier for you?

e. Are there resources you would recommend future researchers provide to community health centers?

f. Would you be interested in participating in this type of research again? Why or why not?

4. Do you have any other comments or thoughts about participating in the ASIST study?

That wraps up the questions I have for you. Is there anything that we didn’t talk about that you would like to tell us?

Lastly, I would like to gather a few pieces of demographic information from you:

What is your title at *health center name*?

How long have you been employed at *health center name*?

What is your primary language?

What is your level of education?

What is your race/ethnicity?

Thank participant.

## Abbreviations

CHC: Community health center; IRB: Institutional review board.

## Competing interests

Only one author has a potential competing interest to report. Dr. Paula Johnson is a consultant to Medco Health Solutions, and is an Independent Director of West Pharmaceutical Services and holds stock of less than one percent in the company. All other authors declare that they have no competing interests.

## Authors’ contributions

HER conceived of the study, acquired the data, analyzed and interpreted the data, drafted and revised the manuscript. KNG interpreted the data and drafted and revised the manuscript. CRC conceived of the study, analyzed and interpreted the data, revised the manuscript, and provided study supervision. LRC acquired the data, and analyzed and interpreted the data. JG provided primary qualitative analysis, coding and interpretation of the data. DAT acquired the data, analyzed and interpreted the data and provided critical revision of the manuscript. MJO and PSO provided critical revision of the manuscript. PAJ conceived of the study, revised the manuscript, obtained funding and provided senior study supervision. All authors read and approved the final manuscript.
